# Impact of delays in radiotherapy of head and neck cancer on outcome

**DOI:** 10.1186/s13014-020-01645-w

**Published:** 2020-08-20

**Authors:** Barbara Žumer, Maja Pohar Perme, Simona Jereb, Primož Strojan

**Affiliations:** 1 Department of Radiation Oncology, Institute of Oncology Ljubljana, Zaloška 2, SI-1000 Ljubljana, Slovenia; 2 Institute of Biostatistics and Medical Informatics, Faculty of Medicine, University of Ljubljana, Ljubljana, Slovenia; 3 Department of Radiology, Institute of Oncology Ljubljana, Ljubljana, Slovenia; 4 Chair of Oncology, Faculty of Medicine, University of Ljubljana, Ljubljana, Slovenia

**Keywords:** Head and neck cancer, Radiotherapy, Waiting time, Treatment delay, Outcome

## Abstract

**Background:**

In head and neck cancer (HNC), the relationship between a delay in starting radiotherapy (RT) and the outcome is unclear. The aim of the present study was to determine the impact of the amount of time before treatment intervention (TTI) and the growth kinetics of individual tumors on treatment outcomes and survival.

**Methods:**

Two hundred sixty-two HNC patients with 273 primary tumors, treated with definitive (chemo) RT, were retrospectively analyzed. The TTI was defined as the time interval between the date of histopathologic diagnosis and the first day of the RT course. Volumetric data on 57 tumors were obtained from diagnostic and RT planning computer tomography (CT) scans in order to calculate the tumor growth kinetic parameters.

**Results:**

No significant association between locoregional control or cause-specific hazards and TTI was found. The log hazard for locoregional recurrence linearly increased during the first 40 days of waiting for RT, although this was not significant. The median tumor volume relative increase rate and tumor volume doubling time was 3.2%/day and 19 days, respectively, and neither had any impact on locoregional control or cause-specific hazards.

**Conclusion:**

The association between a delay in starting RT and the outcome is complex and does not harm all patients waiting for RT. Further research into imaging-derived kinetic data on individual tumors is warranted in order to identify patients at an increased risk of adverse outcomes due to a delay in starting RT.

## Background

With 835,000 new cases and 431,000 deaths reported in 2018, head and neck cancer (HNC) is the eighth most common and most lethal cancer in men worldwide [1]. In addition to surgery and systemic therapy, radiotherapy (RT) is one of the cornerstones for treatment of this cancer. Owing to the rising cancer incidence rate in ageing populations and the widening list of indications for irradiation, the demands for RT have increased dramatically over the past decades [2, 3]. The increasing complexity of pre-treatment diagnostics and RT technology has led to delays in treatment decision-making and the reduction in linear accelerator throughput that has resulted in a significant imbalance between the demand for RT and the availability of RT capacities in many publically funded health systems; this is also the case in Slovenia [4, 5].

Due to obvious ethical reservations, the only way to study the impact of delays in starting RT on treatment outcomes are retrospective, observational analyses of cohorts from different institutions or countries [6]. Intuitively, one would expect that the prolongation of the time taken before treatment intervention (TTI) is harmful to patients. Both the likelihood of tumor growth and the acquisition of a metastatic phenotype increases as a function of time [7]. Furthermore, advanced tumors are more difficult to treat than smaller tumors [8].

Indeed, a systematic review of pertinent literature from the period 1975–2005 by Chen et al. showed an increase in the risk of local HNC recurrence of 3.7% for every month of delay in definitive RT [9]. However, certain studies included in Chen’s meta-analysis, and also some more recent reports, negated the association between the delay in definitive RT and the increased risk of treatment failure [10–18]. Several different biases, inherent in retrospective analyses, either related to the quality of diagnostic procedures and treatment or to the inhomogeneity of the studied population, as well as a selection bias (i.e., patients with fast tumor progression or a higher burden of symptoms receive priority in treatment) and significant variability in the kinetics of individual HNC cases, may abolish the effect of TTI in outcomes [19–25]. However, if patients with advanced or fast-growing tumors have to wait longer, the magnitude of this effect may be overestimated [26]). Furthermore, no compelling relationship between treatment delay and prognosis was found in some other cancer types [27–32].

In order to determine what would be an acceptable TTI in HNC patients treated with definitive RT or concurrent chemoradiation, we aimed to analyze the impact of TTI and growth kinetics of individual tumors on the occurrence of local/regional failure, distant metastasis, and survival in the present study of a cohort of Slovene patients with HNC.

## Methods

In a retrospective study, patients with oral cavity, oropharyngeal, hypopharyngeal, or laryngeal squamous cell carcinoma (SCC) who were treated with curative-intent definitive RT, with or without concurrent chemotherapy between January 2004 and December 2007 at the Institute of Oncology, Ljubljana, Slovenia, were included. Patients with T1N0 or T2N0 glottic cancer were left out of this cohort. The 2004–2007 period was chosen because of fluctuations in the waiting time for irradiation as a result of intensive renovations and expansion in the Department of Radiotherapy that took place over this time span. From patients’ medical and RT charts, we collected information on clinical (gender, age, onset of symptoms, date and type of disease recurrence, and death), tumor (histology, site of origin, TNM stage), and treatment characteristics (RT technique, regimen and dose, duration of RT, type of concurrent chemotherapy [CCT]), and the number of cycles administered). The TNM stage was determined according to the 7th edition of the UICC classification system.

For analysis of the impact of tumor growth kinetics on treatment outcomes, the volumes (mL) of primary tumors and neck nodal metastases, as marked on diagnostic and RT planning computer tomography (CT) scans, were compared. Patients with the same basic clinical, disease and treatment characteristics, as indicated above but with both sets of CT scans available, were selected for this part of the study. Diagnostic CT scans were performed through the acquisition of 2 mm thin CT sections, whereas planning CT scans had a slice thickness of 3 mm, both with intravenous iodine contrast enhancements. Sets with extensive artefacts were excluded. For the purposes of RT planning, patients were positioned supine on the flat tabletop and a five-fixation point thermoplastic mask was used. Lymph nodes were considered positive if the smallest diameter was more than 1 cm and/or the necrotic center or extracapsular extension was seen. If available, segmentation was guided by magnetic resonance imaging (MRI) sets and the resulting contours around the primary tumors and metastatic neck nodes represented a consensus between two radiation oncologists and a radiologist. Volumes of primary tumors and metastatic neck nodes were separately calculated by a computer software program used for RT planning (XiO, Computerized Medical Systems Inc., St. Louis, USA; Eclipse Varian Medical Systems Inc., Palo Alto, USA). The end points in this part of the study were changes in the tumor/nodal volume and TNM stage, the calculation of the primary tumor volume doubling time, and their impact on the treatment outcome.

### Statistics

The study protocol was approved by the Republic of Slovenia National Medical Ethics Committee (No. 0120–573/2017/4). For retrospective studies, a written consent is deemed unnecessary according to national regulations.

Basic descriptive statistics were reported with means, standard deviations and ranges for numerical variables, and as percentages for categorical variables. In patients with two simultaneous HNCs, some characteristics were reported in regards to patients, while others were reported in regards to tumors.

The survival curves were computed using the Kaplan-Meier estimator and the Aalen-Johansen estimator was used to estimate the cumulative probabilities of competing risks. The effect of covariates was analyzed using a multiple Cox regression analysis. With all the analyses, the data were censored at a five-year follow-up.

When focused on the survival of patients, the analyses were completed with patients as the units and the time was measured from the first day of therapy until death. The overall survival (OS, regardless of the cause of death) and the absolute risk (cumulative probability) of dying due to index cancer were reported. In the Cox regression, only the index cancer deaths (cause-specific hazard, CSH) were considered to be events of interest.

In the analyses where locoregional control (LRC) was of primary interest, the calculations were performed in regards to tumors, excluding the non-responders to RT (i.e., those with residual local or regional tumors at 10–12 weeks after RT completion). For the latter group, we concluded that it is the radio-resistance of tumor cells that are responsible for the persistence of the disease after therapy and not that patients had to wait for RT. The follow-up time was calculated from the last day of therapy. The estimated cumulative probability of local and/or regional recurrence, distant metastases, LRC (probability of being still alive and without local and/or regional recurrence), and disease-free survival (DFS; probability of being still alive and without events: locoregional and distant failure and deaths were the events of interest) were reported. All the analyses were conducted in regards to the tumors as independent units (this assumption was checked in the sensitivity analysis and allowed for gamma frailty).

The assumptions of the Cox regression were checked: a non-linear effect (a spline with 2 degrees of freedom) was allowed for the numerical variables and the proportional hazards assumption was tested using Schoenfeld residuals.

The TTI was defined as the time interval between the date of histopathologic diagnosis and the first day of the RT course. Tumor growth kinetics was expressed as the tumor volume relative increase rate (per day) and as the tumor volume doubling time (in days).

The tumor volume relative increase rate (per day) was calculated as:

$$ {\left(\frac{V\left({T}_2\right)}{V\left({T}_1\right)}\right)}^{\frac{1}{T_2-{T}_1}} $$

where V(T1) = gross tumor volume at time 1 (T1), i.e., on diagnostic CT scans; and V(T2) = gross tumor volume at time 2 (T2), i.e., on planning CT scans. It was reported as the percentage increase (1.01 was reported as 1% per day). The tumor volume doubling time was calculated as:

$$ \frac{\mathit{\ln}(2)\left({T}_2-{T}_1\right)}{\mathit{\ln}\frac{V\left({T}_2\right)}{V\left({T}_1\right)}} $$

Since a one-to-one relationship existed between the two, only the tumor volume relative increase rate (which requires no extrapolation) was considered for modelling.

All analyses were performed using R statistical software (version 3.4.1) and a *p*-value below 0.05 was considered statistically significant.

## Results

### Impact of time to treatment initiation

**Fig. 1:**
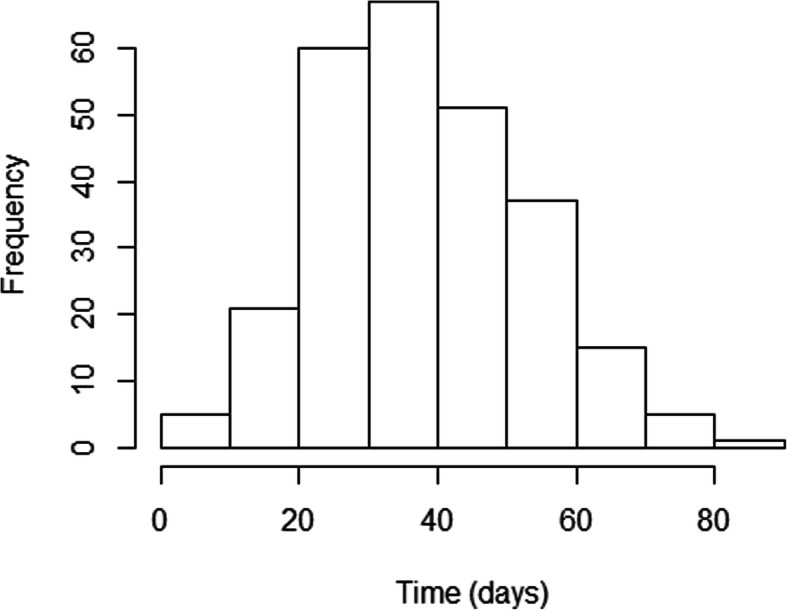
Distribution of TTI

Between 2004 and 2007, 262 patients with 273 oral cavity, oropharyngeal, hypopharyngeal, or laryngeal primary SCCs were treated with definitive (chemo) radiotherapy with curative intent. There were 227 men and 35 women, aged 38–89 years (mean: 60). The majority of tumors originated in the oropharynx (133 tumors in 130 patients) and were TNM stage IV (163 tumors in 157 patients). The TTI ranges from 7 to 90 days with mean 35.6 days. The distribution of the TTI is only slightly asymmetric with median 36 and interquartile range (IQR) 28–48.8 (Fig. 1). Patients were irradiated with 2-dimensional (46, 17.6%) technique or 3-dimensional conformal isocentric technique (216, 82.4%) to the median RT dose 70 Gy (IQR 70–70) delivered in 2 Gy daily fractions (IQR 2–2). Concurrently to RT, 116 patients (42.5%) received chemotherapy, consisted of platin-based mono-chemotherapy (81 patients) or mitomycine C-bleomycin combination (35 patients). The characteristics of patients and their tumors are shown in Table 1.

**Table 1:** Clinical characteristics of patients and their tumors

Characteristics	No. (%)
Gender	
Female	35 (13.2%)
Male	227 (86.8%)
Age (years)^a)^	60.6 ± 9.9 (38.9–89.3)
Tumor location^b)^	
Oral cavity	15 (5.5%)
Oropharynx	133 (48.7%)
Hypopharynx	53 (19.4%)
Larynx	72 (26.4%)
Tumor stage^b)^	
Stage I	12 (4.4%)
Stage II	50 (18.3%)
Stage III	48 (17.6%)
Stage IV	163 (59.7%)
Treatment^b)^	
Radiotherapy	157 (57.5%)
Concurrent chemoradiotherapy	116 (42.5%)
RT duration (days)^a)^	50.2 ± 6.7 (35–80)
Time to treatment intervention^a)^	35.6 ± 14.9 (7–90)

^a)^ mean ± SD (range)

^b)^ Eleven patients had two simultaneous primary tumors

#### Treatment outcome and survival

Clinical and/or radiologic assessment at 10–12 weeks post-therapy confirmed disease persistence locally and/or regionally in 66 cases (i.e., in 65 patients), and these patients were excluded from further analyses of LRC and DFS. Thus, 197 patients with 207 tumors were analyzed, and during follow-up, a local and/or regional relapse was recorded in 70 cases (33.8%) with a median time from treatment completion of 9 months (range: 6–18 months). The two- and five-year probability of local recurrence after the end of treatment was 20.3 and 24.2%, respectively, whereas the regional relapse was 12.6 and 15.5%, respectively. After three-years post-therapy, we only observed a small increase (i.e., 2%) in local recurrence probability, whereas the probability of regional relapse was stable after the third year follow-up. The probability of occurrence of distant metastases at 2 years was 8.7% and at 5 years it was 10.1%. At the second- and fifth-year follow-up, the LRC was 52.7% (95% confidence interval [CI], 46.3; 59.9) and 31.4% (95% CI, 25.7; 38.4), respectively, and the DFS was 50.2% (95% CI, 43.9; 59.9) and 31.4% (95% CI, 25.7; 38.4), respectively.

In the fifth year post-treatment, 187 out of 262 (71.4%) patients had died. The index cancer was the reason in 132 (70.6%) of the cases. During the first 2 years post-therapy, the probability of cancer-related death steeply increased from zero to 40%, but later, the changes were less pronounced. At the second and fifth year post-treatment, the OS, independent of the cause of death, was 51.5% (95% CI, 45.8; 57.9) and 28.6% (95% CI, 23.6; 34.7), respectively.

#### Effect of covariates

**Fig. 2:**
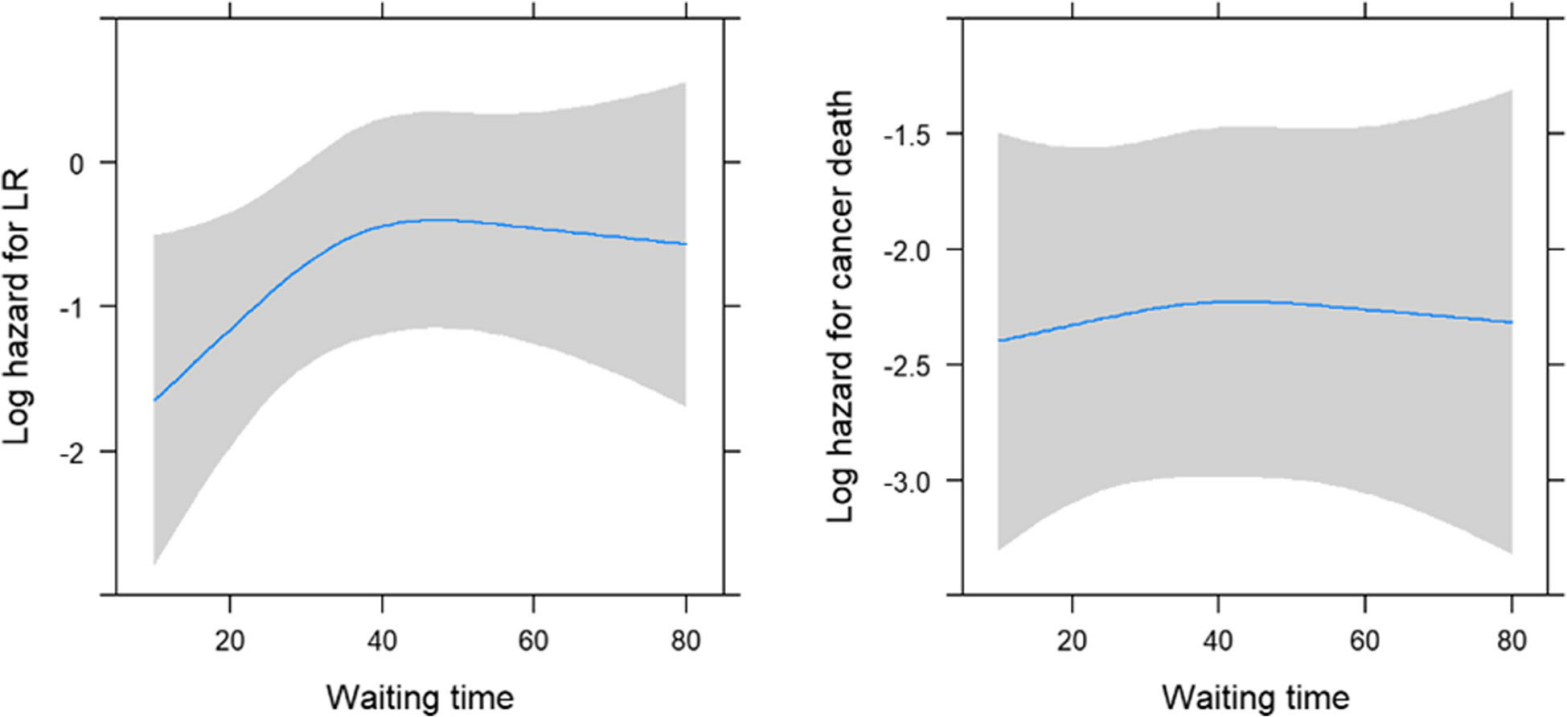
Trend of the hazard for locoregional recurrence (**a**) and for index cancer-specific death (**b**)

In the univariate analysis, the following parameters were tested for LRC and CSH: age, gender, stage of disease, type of treatment (RT vs. RT + CCT), and TTI for RT (as continuous variable). Results are presented in Table 2. The same parameters were also included in the multivariate model (Table 3). The occurrence of locoregional recurrence was significantly related to the disease stage (*p* = 0.002), whereas the relationship with age was only of marginal significance (*p* = 0.075; higher age had a lower hazard). No significant association with the type of treatment could be found (*p* = 0.344) (Table 3). The log hazard for locoregional recurrence seemed to linearly increase during the first 40 days of waiting for RT, although the association between the hazard and TTI was insignificant, regardless of whether we made an allowance for nonlinearity or not (Fig. 2a).

**Table 2:** Effect of covariates on locoregional control and index cancer-specific hazard (*N* = 197 patients with 207 tumors): an univariate analysis

Parameter	HR	95% CI	*P*-value
*Locoregional control*			
TNM (III vs. I-II)	0.855	0.378–1.934	0.706
TNM (IV vs. I-II)	2.265	1.276–4.021	0.005
Age	0.969	0.944–0.994	0.015
TTI	1.013	0.997–1.028	0.104
Gender	0.476	0.191–1.183	0.110
Therap (CCT vs. RT)	1.370	0.857–2.189	0.188
*Cause specific hazard*			
TNM (III vs. I-II)	0.855	0.378–1.934	0.026
TNM (IV vs. I-II)	2.265	1.276–4.021	< 0.001
Age	0.979	0.961–0.997	0.024
TTI	1.003	0.991–1.014	0.631
Gender	0.827	0.483–1.416	0.489
Therapy (CCT vs. RT)	1.234	0.877–1.736	0.228

*CI* Confidence interval; *TTI* Time to treatment intervention;

*CCT* Concurrent chemoradiotherapy; *RT* Radiotherapy

**Table 3:** Impact of TTI on locoregional control and index cancer-specific hazard (*N* = 197 patients with 207 tumors): a multivariate analysis

Parameter	HR	95% CI	*P*-value
*Locoregional control*			
TNM (III vs. I-II)	0.843	0.369–1.925	0.685
TNM (IV vs. I-II)	2.320	1.211–4.444	0.011
Age	0.976	0.950–1.002	0.075
TTI	1.012	0.998–1.027	0.094
Gender	0.501	0.201–1.249	0.138
Therapy (CCT vs. RT)	0.768	0.444–1.328	0.344
*Cause specific hazard*			
TNM (III vs. I-II)	3.203	1.286–7.976	0.012
TNM (IV vs. I-II)	13.223	6.007–29.108	< 0.001
Age	0.985	0.965–1.005	0.133
TTI	1.001	0.990–1.012	0.844
Gender	0.907	0.529–1.556	0.724
Therapy (CCT vs. RT)	0.534	0.369–0.774	0.001

*CI* Confidence interval; *TTI* Time to treatment intervention;

*CCT* Concurrent chemoradiotherapy; *RT* Radiotherapy

The hazard of dying due to the index cancer (i.e. CSH) was found to be favorably associated with a lower disease stage (*p* < 0.001) and the addition of CCT to RT (*p* = 0.001), whereas age, gender, and TTI did not reach the level of statistical significance (Table 3, Fig. 2b).

#### Assumptions and sensitivity analysis

Proportional hazards (for all included variables) and the linearity assumption (for age) were analyzed and presented graphically. Some indication of non-proportional hazards could be found in gender, but allowing for non-proportional hazards did not change the interpretation of the other covariates. As a part of the sensitivity analysis, the assumption of the dependence of the tumors belonging to the same individual was relaxed, but no changes in the interpretation of the results could be observed. As an additional confounder, the time-dependent covariate “duration of RT” was considered, but its effect did not prove to be important.

### Impact of tumor growth kinetics

Diagnostic and planning CT scans (median interval: 19 days, range: 3–108 days) were available from 52 patients; five of these patients had two primary tumors. The majority of patients were males (43, 82.7%), with a mean age of 61 years (range: 43.5–81.5), and the patients had primary tumors located in the oropharynx (Table 4).

**Table 4:** Characteristics of patients with available diagnostic and planning CT scans

Characteristics	No. (%)
Gender	
Female	9 (20%)
Male	36 (80%)
Age (years)^a)^	60.47 (43.5–81.5)
Tumor location^b)^	
Oropharynx	35 (61.4%)
Hypopharynx	3 (5.3%)
Larynx	19 (33.3%)
Stage^b)^	
Stage I	5 (8.8%)
Stage II	2 (3.5%)
Stage III	14 (24.6%)
Stage IV	36 (63.2%)
Therapy^b)^	
Radiotherapy	9 (20%)
Concurrent chemoradiotherapy	36 (80%)
Tumor volume relative increase rate (%/day)^a)^	1.03 (0.99–1.41)
Tumor volume doubling time (days)^a)^	19 (2–659)

^a)^ mean ± SD (range)

^b)^ Five patients had two simultaneous primary tumors

When two CT sets were compared, the original T-stage of the primary tumor was increased (i.e., upgraded) in two cases and the nodal stage was increased in six patients. Due to the limited number of N-positive cases (*n* = 34, 59.6%), only volumes of primary tumors were compared between the two CT sets for the calculation of the tumor volume relative increase rate (% per day) and tumor doubling time. The absolute increase in tumor volume ranged from − 0.06 to 3.87 cm^3^ per day (median: 0.29); no increase in volume was observed in five patients. CT scans in these five patients were taken from 4 days to 35 days apart (median: 19 days). The median tumor volume relative increase rate was 3.2% per day (median, 1.03; range, 0.99–1.41) and the median tumor volume doubling time was 19 days (range, 2–659 days). No difference was observed when these two parameters were compared between different tumor stages.

#### Treatment outcome and survival

Among the 57 tumors, the time taken for local and/or regional relapse and the occurrence of distant metastases were assessed for 45 tumors in which treatment resulted in clinical/radiological eradication of the disease at 10–12 weeks post-therapy. Local and/or regional relapse was recorded in nine (20%) cases with a median time from treatment completion of 9 months (range: 7–15 months). The two and five-year probability of local recurrence after the end of the treatment was 11.1 and 13.7%, respectively, whereas for the regional relapse, it was 6.7 and 9.2%, respectively. At 3 years post-therapy, only an increase in the local recurrence probability of 2% was observed, whereas the probability of regional relapse remained stable. The two- and five-year probability of distant metastases was 4.4%. At two and 5 years, the LRC was 71.1% (95% CI, 59; 85.7) and 62.2% (95% CI, 48.8; 79.3), respectively, and the DFS was 68.9% (95% CI, 56.6; 83.8) and 62.4% (95% CI, 49.1; 79.5), respectively.

In the 5 year-period after the start of treatment, 24 out of 52 (64.2%) patients died: the index cancer was the reason in 18 (75%) cases. At two and 5 years post-treatment, the OS rates were 65.4% (95% CI, 53.7; 79.7) and 51.3% (95% CI, 38.7; 68.1), respectively.

#### Effect of covariates

**Fig. 3:**
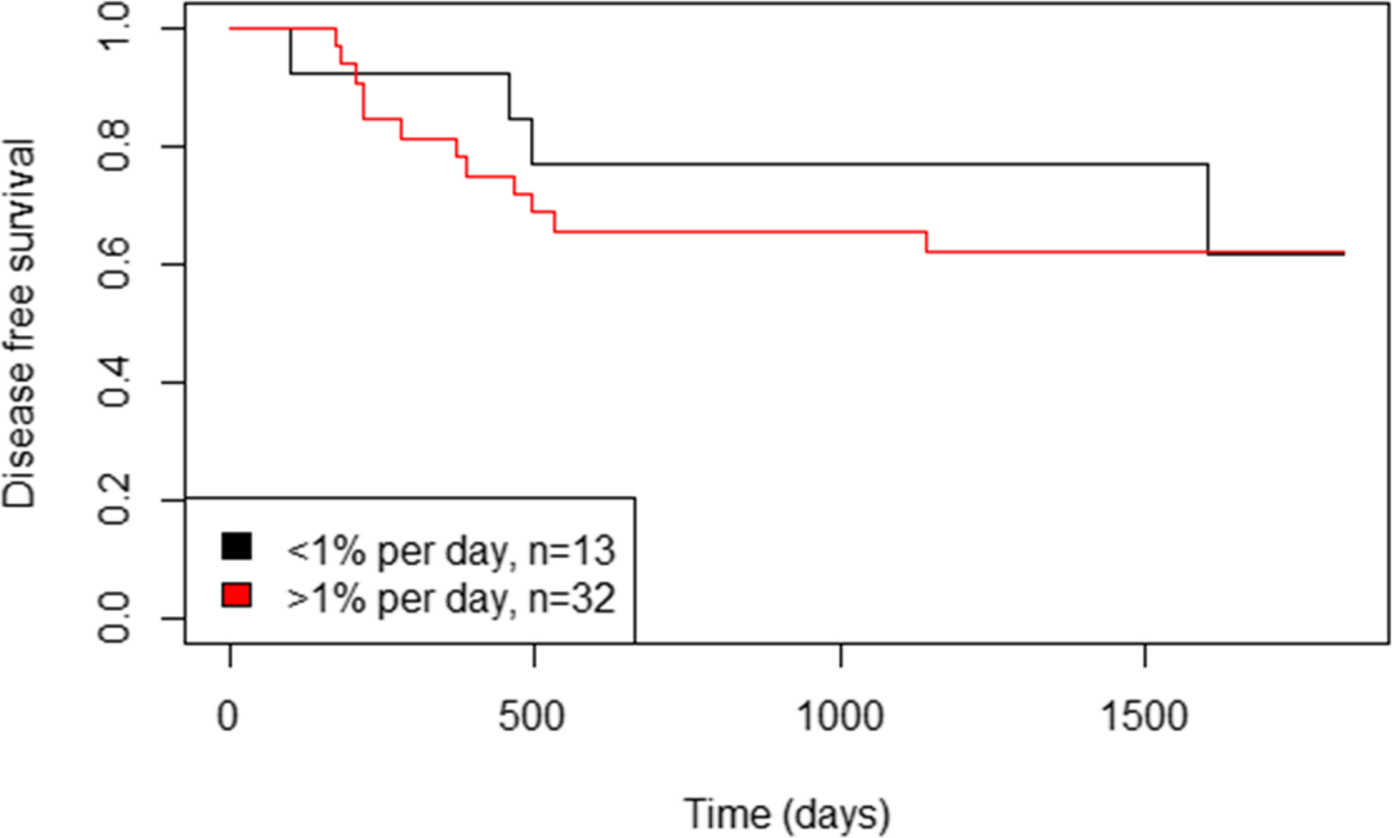
Impact of primary tumor volume relative increase rate to disease-free survival in 45 patients with no residual disease at 10–12 weeks post-therapy

Due to the low number of events (local and/or regional recurrence: nine events; cancer-specific deaths: 18 events), only univariate regression models were fitted. The following covariates were tested in the models: overall disease stage, initial tumor volume, tumor doubling time, and relative increase rate (%/day) of primary tumor volume. Measures of tumor kinetics did not show any impact on LRC or CSH; only an inverse relationship between the initial primary tumor volume and CSH was observed (HR = 1.02 for index cancer-specific death per every 1 cm^3^ increase in the volume of primary tumors) (Table 5). There was no difference in the value of DFS between patients with a primary tumor volume relative increase rate < 1%/day and > 1%/day (*p* = 0.755, Fig. 3).

**Table 5:** Effect of stage and tumor kinetics on locoregional control and overall survival

Parameters	Locoregional control			Cause specific hazard		
	HR	95% CI	*P*-value	HR	95% CI	*P*-value
TNM stage (IV vs. I-III)	2.73	0.57–13.18	0.210	2.46	0.82–7.41	0.110
V_TU_ (per cm^3^)	1.02	0.99–1.04	0.168	1.02	1.01–1.00	0.005
V_TU_ relative increase rate (per %/day)	0.99	0.89–1.09	0.811	0.97	0.88–1.06	0.465
V_TU_ relative increase rate (≤1% vs. > 1%/day)	1.52	0.31–7.31	0.603	1.48	0.49–4.47	0.483

*V*_*TU*_ Volume of primary tumor

## Discussion

While it is intuitively anticipated and confirmed by the results of the meta-analysis that a delay in starting RT would have a negative impact on the treatment of patients with HNC, this association was not confirmed in our study [9]. We only found a statistically insignificant upward trend in the risk of locoregional recurrence for the first 40 days of RT delay. In addition, the differences in the growth kinetics between individual tumors, which were studied in a smaller group of patients, were considerable but did not appear to be of significance for the prediction of treatment outcomes.

Obviously, the relationship between TTI and disease prognosis in patients with HNC is more complex than it might seem at first glance. The first negative impact of waiting for treatment to begin is the risk that the tumor will increase in size and/or metastasize during this time, making it harder to treat or resulting in it becoming untreatable [7, 8]. However, the time for a tumor to grow is only one of the factors that determines prognosis and the absence of a statistically significant association between TTI and the treatment outcome in our study (and many other reports) is thus not surprising [10–18]. Moreover, no obvious methodological differences could be found between these (negative) studies and the positive studies that confirmed the association between TTI and treatment outcomes [33–35]. In both groups, there are individual studies that have similar sample sizes, tumor site/stage mix, and periods covered, speaking in favor of comparable quality of diagnostics, therapy, and statistics across the studies.

On the contrary, in more recently reported analyses of the national cancer registries data, an adverse effect of waiting for radiotherapy was clearly established [36–38]. However, the results from this type of analysis should be taken with caution, not only due to the limitations inherent in the tumor registry data (unmeasured confounding, selection bias, incomplete data, and coding errors), but also because the effect of delaying RT on cancer-specific outcomes was not evaluated. In addition, patients irradiated in postoperative and definitive settings were not analyzed separately. Overall, the research methodology and interpretation used in these studies were criticized and the magnitude of the effect that they supposedly demonstrated was questioned [39].

In the present study, a linear increase in the log hazard for locoregional recurrence was found during the first 40 days of waiting for RT, although it was not statistically significant. It is possible that unknown confounders that were not accounted in our analysis (e.g., tumor growth kinetics) reduced the statistical power of the TTI. The results from Fortin et al. and Naghavi et al., who reported on the increased risk of locoregional failure with TTI > 40 days and > 45 days, respectively [33, 35], are the most comparable to our own. However, optimal TTI thresholds identified in different studies showed considerable variations, pointing to the uncertainty of such calculations and their dependence on the characteristics of the analyzed population [34, 36–38]. The possible role of classical prognostic factors, such as location of primary tumor, disease stage, and addition of CCT is not expected to be relevant in this respect, as in our and other similar studies the statistical significance of TTI was verified by multivariate analysis.

The effect of TTI on treatment outcomes, however, is not only conditioned by the duration of waiting for RT, but also on the rate of tumor growth. In HNC, a vast heterogeneity in tumor cell kinetics has been observed, conditioned by the local milieu from which it arises, which differs from patient to patient [8, 40, 41]. Historically, different methods were explored to evaluate tumor cell kinetics, but did not succeed in providing clinically relevant kinetic parameters [42, 43]. More recently, a comparison of two sets of imaging data, acquired at two different time points, was successfully employed for this purpose. Usually, volumetric data are extracted from diagnostic and RT-planning CT scans for the calculation of different parameters reflecting the rate of tumor growth, e.g., (tumor volume) doubling time or absolute/percentage tumor volume increase per day. Despite some differences in the calculation methodology across studies, a large variation in the individual values of kinetic parameters was seen in all of them, including ours, indicating that all of the studied populations represent a unique mix of slow and fast growing tumors [19–25]. Among our patients, five had no measurable increase in primary tumor volume even when the interval between CT scans was up to 35 days. The inverse relationship between kinetic parameters, determined by the comparison of two CT datasets, and treatment outcomes was implied or even confirmed in several smaller studies, pointing to potential clinical utility of imaging-derived kinetic data [21–25]. In our group, however, no such association was found: a small number of patients and – in contrast to other studies – the inclusion of different tumor sites could contribute to the negative result, as well as differences in the changes in kinetic properties triggered by RT/CCR in individual tumors [44].

Tumor radiosensitivity may also influence the effect of TTI on outcome. While to some extent it may be evaluated before RT begins (e.g., by molecular profiling, identification of hypoxic cells, and the determination of HPV status in oropharyngeal primary tumors), in daily clinical practice it is usually not considered when planning treatment [45, 46]. In order to diminish the impact of intrinsic tumor radiosensitivity, the patients in our study with residual disease at 10–12 weeks post-RT were excluded from the analysis of LRC. We hypothesized that the inability to achieve a complete tumor response to (chemo) radiation was due to the radiobiological characteristics of the disease and not the delay in starting RT. However, the exclusion of these patients from the analysis did not affect the end results.

Our study has weaknesses, which are mostly due to its retrospective nature. However, for obvious ethical reasons, this is an inevitable feature of studying delays in the initiation of cancer therapy. We are aware that there is always some doubt as to the accuracy of the diagnostic procedures, the staging, the quality of planning and the implementation of RT in the case of retrospective research. Nonetheless, the same restraint exists in the case of other similar studies and even more so in the case of analyses of cancer registry datasets. An important feature of our data set is that no patients were lost during the follow-up and, in our opinion, is free from many hidden biases that larger studies inevitably bring with. Since almost half of our patients had oropharyngeal carcinoma, missing information regarding the p16 or human papillomavirus (HPV) tumor status could be of importance. However, in the cohort of oropharyngeal cancer patients treated at our institution between 2007 and 2008, only 20.2% had a HPV-related tumor [47]. Thus, we reasonably assume that the impact of p16/HPV tumor status on the study results was negligible. In addition, in HPV-positive cases, Perni et al. found no significant association between tumor growth velocity (calculated from serial pre-treatment CT scans), local and distant control, or OS, and the same was reported by Chu et al. who measured metabolic growth velocity using pre-treatment PET-CT scans [23, 48].

Other drawbacks to consider include technical limitations in CT scan acquisition, the accuracy of presentation of actual tumor volume on CT images, and the precision of the delineation of gross tumor volume. To minimize errors in the estimation of comparative tumor volumes, only high-quality pairs of CT images were selected for the analysis of tumor kinetics. In addition, physical findings documented in clinical records and, when available, diagnostic MRIs were used for this purpose and labelled tumor volumes represented a consensus between two experienced radiation oncologists and a radiologist, all dedicated to HNC management. Like many other studies [10–18, 33–36], the comorbidity burden was not registered in our patients, although it should be taken into account when assessing survival outcomes [49]. Finally, lags in the pre-biopsy period were not addressed in our study, including patient delay and delays in referral and diagnostics, which may add significantly to the total TTI. These are also costly and potentially fatal, but can be successfully reduced by effective coordination between providers [50–52].

## Conclusions

The relationship between TTI and treatment outcomes is multifaceted, so the controversy of published results is not surprising. In this study, we found that delays in the onset of RT do not harm all patients. As TTI is a problem in many public health systems, further research is warranted and should focus on two areas: evaluating large population surveys with high-quality data and treatment-related outcomes (not just OS), and the prognostic relevance of imaging-derived kinetic data of individual tumors (which appeared promising in several smaller and statistically underpowered studies) in order to obtain a tool to identify patients at increased risk of treatment failure due to delays in starting RT. In a situation without clear knowledge to whom waiting for irradiation is harmful, the only possible recommendation could be that the waiting time for RT should be “as short as reasonably achievable” (ASARA) [53].

## Data Availability

The datasets analyzed during the current study are available from the corresponding author on reasonable request.

## Abbreviations

HNC: Head and neck cancer
RT: Radiotherapy
TTI: Time to treatment intervention
SCC: Squamous cell carcinoma
CCT: Concurrent chemotherapy
CT: Computer tomography
MRI: Magnetic resonance imaging
OS: Overall survival
CSH: Cause-specific hazard
LRC: Locoregional control
DFMS: Distant metastasis-free survival
DFS: Disease-free survival
IQR: Interquartile range
CI: Confidence interval
HPV: Human papillomavirus
